# Short-term and long-term outcomes in rheumatoid arthritis patients following percutaneous coronary intervention: A systematic review and meta-analysis

**DOI:** 10.1097/MD.0000000000044458

**Published:** 2025-09-12

**Authors:** Sulochana Khadka, Bibek Timilsina, Akshata Ammembal, Faisal Chowdhury, Karun Suwal, Bibek Shrestha, Bishow Nath Adhikari, Bimarsh Acharya, Rabin Baniya, Sushil Gyawali, Sonali Kumari Shah, Rosna Thapa

**Affiliations:** a Department of Medicine, UPMC Harrisburg, Harrisburg, PA; b Department of Medicine, Virtua Our lady of Lourdes Hospital, Camden, NJ; c Department of Medicine, Kasturba Medical College, Manipal, India; d Department of Psychiatry, Chittagong Medical College Hospital, Chattogram, Bangladesh; e Department of Orthodontics, Nepal Medical College, Kathmandu, Nepal; f Institute of Medicine, Tribhuvan University Teaching Hospital, Kathmandu, Nepal; g Department of Pediatrics, Kanti Children’s Hospital, Maharajgunj, Kathmandu, Nepal; h Department of Medicine, Kist Medical College and Teaching Hospital, Lalitpur, Nepal; i Department of Medicine, Nepal Medical College and Teaching Hospital, Kathmandu, Nepal; j Department of General Surgery, Tribhuvan University Teaching Hospital, Kathmandu, Nepal; k Department of Hospital Administration, Chitwan Medical College, Chitwan, Nepal; l Department of Surgery, Nepal Cancer Hospital and Research Center. Lalitpur, Nepal.

**Keywords:** angiogram, coronary intervention, PCI, rheumatic arthritis

## Abstract

**Background:**

Rheumatoid arthritis (RA) is associated with an elevated risk of cardiovascular disease and necessitates repeat revascularization procedures, including percutaneous coronary intervention (PCI). However, extensive data on outcomes following PCI in this cohort remain scarce. This systematic review and meta-analysis sought to evaluate the short- and long-term cardiovascular outcomes in RA patients following PCI.

**Methods:**

We conducted a search of PubMed, Embase, Google Scholar, and Cochrane Central for studies published until October 2024 that compared RA and non-RA cohorts’ post-PCI. The primary outcomes encompassed major adverse cardiovascular events, myocardial infarction, repeat revascularization, and overall mortality. The pooled odds ratio (OR) with 95% confidence intervals was computed utilizing random-effects models. A sensitivity analysis was conducted using a leave-one-out meta-analysis.

**Results:**

Our search identified 9 qualifying studies, encompassing nearly 1 million patients (174,229 with RA and 9771,911 without RA). Individuals with RA exhibited a markedly elevated risk of short-term stroke compared to non-RA patients (OR: 0.81, 95% CI: 0.6–1.02). Long-term follow-up found an elevated risk of myocardial infarction (OR: 1.08, 95% CI: 1.01–1.16), stroke (OR: 1.09, 95% CI: 1.07–1.11), major adverse cardiovascular events (OR: 1.12, 95% CI: 0.99–1.24), and repeat revascularization (OR: 1.09, 95% CI: 1.07–1.11) among patients with RA. The sensitivity analysis revealed no significant difference, even after the exclusion of each study.

**Conclusion:**

This comprehensive meta-analysis revealed that patients with RA have markedly poorer clinical outcomes post-PCI, particularly in the long term. The results underline the necessity for tailored peri-procedural approaches and ongoing monitoring in RA patients.

## 1. Introduction

Rheumatoid arthritis (RA) is a chronic autoimmune inflammatory disorder that predominantly presents as symmetric polyarthritis, while also impacting various organ systems, including the heart, lungs, eyes, skin, digestive system, and central nervous system.^[1]^ Cardiovascular issues constitute the primary cause of mortality in RA patients, representing 39.6% of fatalities, succeeded by respiratory disorders. Moreover, patients with RA have a 50% elevated risk of cardiovascular mortality relative to the general population,^[2,3]^ a risk level comparable to that associated with type 2 diabetes mellitus.^[4]^

The cardiovascular consequences include pericarditis, endocarditis, heart failure, and coronary artery disease.^[5]^ Ischaemic heart disease constitutes the predominant cardiovascular risk in persons with RA. The etiology is a prolonged inflammatory process within the blood vessels, resulting in accelerated atherosclerotic plaque rupture, thrombosis, and subsequent obstruction of the coronary arteries. The systemic inflammation associated with RA also leads to elevated levels of low-density lipoprotein, hence exacerbating this risk.^[6]^ This inflammatory process not only elevates the incidence of these conditions but also adversely affects the prognosis following coronary procedures.^[7–9]^ Percutaneous coronary interventions (PCI), a minimally invasive procedure that involves dilating arteries with balloons and implanting stents, is the most prevalent revascularisation technique^[10]^

Previous studies, including case reports and cross-sectional analyses, have sought to determine the impact of various rheumatological conditions, such as RA, on the prognosis of patients with coronary artery disease following PCI. Many of these studies have concentrated on various rheumatological illnesses, with some examining short-term outcomes and others assessing long-term outcomes.^[11]^ We have compiled and synthesized this information, focusing primarily on RA to determine its correlation with the outcome of patients receiving PCI. Our objective is to conduct a comprehensive study utilizing extensive data from multiple sources to compile robust evidence regarding the impact of rheumatological disorders on PCI prognosis and the factors affecting related outcomes.

## 2. Methods

This study was conducted in accordance with the 2020 PRISMA Guidelines for Systematic Review and Meta-analysis and adhered to established methodologies.^[12]^ Ethical approval was not required as this study was a systematic review and meta-analysis derived from published literature. Informed consent was unnecessary as the study utilized publically accessible data.

### 2.1. Search strategy

We conducted a literature search in PubMed, Embase, Google Scholar, and Cochrane Central for articles from their inception until October 2024. The following keywords and Medical Subject Headings (MeSH) terms were used with “AND” and “OR” operators: “PCIs,” “Coronary Artery Bypass Grafting,” “Revascularization,” “ischemic cardiomyopathy,” “Coronary intervention,” and “Left ventricular systolic dysfunction.” The complete list of terms can be found in File S3, Supplemental Digital Content, https://links.lww.com/MD/P957. Two authors independently reviewed the abstract and title of the articles for eligibility. Any inclusion-related discrepancies were resolved by the first author.

### 2.2. Eligibility criteria

We included studies involving patients aged ≥18 years without any language restrictions, who were undergoing PCI. All observational cohort studies (prospective or retrospective), case-control studies, and randomized controlled trials that reported quantitative data on at least 1 clinical outcome associated with PCI in RA patients were deemed eligible for inclusion. Studies were required to have a follow-up duration of at least 30 days after PCI and to provide data suitable for pooled analysis.

Review articles, case reports, and editorials were considered ineligible. Studies with common data or insufficient data/sample size for statistical analysis were also excluded. Any studies lacking a comparison with a group of non-RA patients who had undergone PCI were excluded from this review.

### 2.3. Outcomes

The primary outcome of this meta-analysis was long-term events including major adverse cardiovascular events (MACE), myocardial infarction (MI), stroke repeat revascularization, cardiovascular mortality and all-cause mortality.

- **MACE**: A composite endpoint typically including MI, stroke, cardiovascular mortality, and the need for repeat revascularization.^[13]^
- **MI**: Irreversible necrosis of heart muscle secondary to prolonged ischemia, commonly known as a heart attack.^[14]^
- **Stroke**: A sudden loss of brain function caused by an interruption of blood flow to the brain, either due to a blockage (ischemic stroke) or rupture of a blood vessel (hemorrhagic stroke).^[15]^
- **Repeat revascularization**: Any procedure to restore blood flow to a blocked artery, typically repeat PCI or coronary artery bypass grafting.^[16]^

The secondary outcomes were short-term events (<30 days post-PCI), including MACE, MI, stroke, revascularization, and all-cause mortality.

### 2.4. Study selection

The preliminary database search using pre-specified keywords yielded 768 articles, of which 14 duplicate studies were excluded. An additional 723 articles were excluded during the initial post-title and abstract screening based on the inclusion and exclusion criteria and the comparison arm. A full-text review was conducted for the remaining 31 studies identified during the search period, of which 22 articles were not retrieved. Studies were excluded at this stage if they had unmatching target populations, were not primary research articles or case reports, or lacked a comparison arm. Consequently, 9 studies that met the eligibility criteria were included in our study (Table 1).

**Table 1:** Baseline characteristics of the patients.

Author	Country	Duration of RA (year)	Mean age RA (year)	Total patients (study total n)	Total RA patients (n)	Male RA n (%)	Assessed outcomes
Lai et al (2015)^[11]^	Taiwan	3	68	171,287	525	236 (45.0)	MACE, long-term revascularization, long-term all-cause mortality, long-term MI, in-hospital mortality
Dawson et al (2020)^[17]^	Australia	NA	68.9	39,335	756	454 (60.0)	MACE, 30-d revascularization, 30-d all-cause mortality, 30-d MI, stroke, 30-d readmission
Varghese et al (2009)^[18]^	Illinois, USA	NA	66.8	487,589	3974	1723 (43.3)	In-hospital mortality, length of stay
Kim et al (2023)^[19]^	Korea	5.9	74.6	74,623	14,074	6283 (44.6)	Long-term all-cause mortality
Martinez et al (2020)^[20]^	USA	NA	68	6,537,832	69,354	30,655 (44.2)	Unspecified complications
Kang et al (2012)^[21]^	Taiwan	NA	68.8	1440	240	132 (55.0)	In-hospital mortality, Readmission
Ha et al (2023)^[22]^	South Korea	5.7	68.6	236,134	34,493	15,962 (46.3)	MACE, long-term and 30-d revascularization, long-term and 30-d all-cause mortality, long-term and 30-d MI and stroke
Alliu et al (2019)^[23]^	USA	NA	NA	51,732	12,933	Specific RA Male % NA (CTD overall: 36.8%)	AKI, Access site complication, VF, CS, Stroke, In-hospital mortality, LOS
Antia et al (2024)^[24]^	USA	NA	68.9	2,346,168	37,880	17,573 (46.4)	Composite major complications (CVA, cardiac arrest, AHF, VA, major bleeding, AKI), Mortality, LOS, Hospital charges

MACE = major adverse cardiovascular events, MI = myocardial infarction, RA = rheumatoid arthritis.

### 2.5. Data extraction and quality assessment

The following data were extracted from all included studies: study type, author, year of publication, number of patients in both groups, age, sex, follow-up period, comorbidities, and primary and secondary outcomes. Two investigators independently appraised the potential risk of bias for randomized controlled trials using Cochrane risk of bias 2 tools.^[25]^ We classified studies as having a high risk of bias, some concerns, or a low risk of bias based on the scores determined by following the risk of bias 2 tool handbook.

### 2.6. Statistical analysis

Baseline continuous variables were summarized as mean with standard deviation, whereas dichotomous variables were described by frequency or percentage. We performed a conventional meta-analysis for primary and secondary outcomes, adopting the DerSimonian and Laird random-effect model for the study variations. Outcomes were reported as pooled odds ratios (OR) and their corresponding 95% confidence intervals. Statistical significance was met if the 95% CI did not cross the numeric “1” and the 2-tailed *P*-value was <.05. The heterogeneity among studies was assessed by the Higgins I-squared (I²) statistical model, with I² values categorized as <25% (low), 25%–50% (moderate), and >50% (high heterogeneity). Sensitivity analysis was performed using a leave-one-out meta-analysis. Publication bias was assessed using the graphical presentation of funnel plot asymmetry (Abadi et al., 2023). All statistical analyses were performed using S.T.A.T.A. version 17.1 (StataCorp, College Station, TX).

## 3. Results

A total of 768 articles were identified using standard electronic databases (PubMed, Google Scholar, Cochrane Library, and Embase). After removing duplicates, title and abstract screening led to the exclusion of many articles, finally yielding 9 articles that met the inclusion and exclusion criteria for full-text review. The epidemiological characteristics of included studies are provided in Table 1.^[11,17–24]^

### 3.1. Quality assessment

All studies had a moderate risk of bias as depicted in Table S1, Supplemental Digital Content, https://links.lww.com/MD/P958.

### 3.2. Meta-analysis

This study revealed that while short-term follow-up outcomes were comparable among RA patients and non-RA patients following PCI, long-term outcomes showed significant differences for certain endpoints.

**Figure 1.**
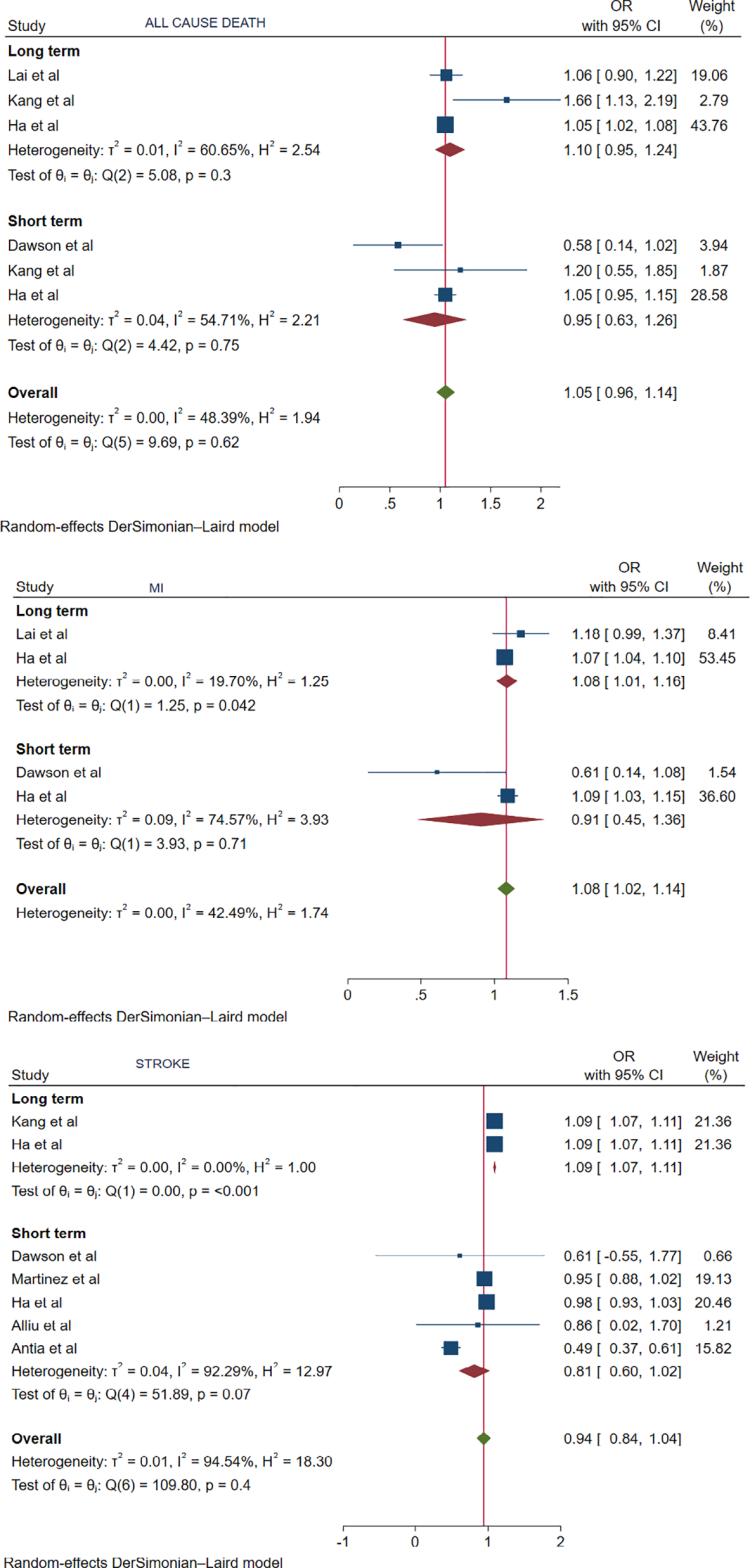
Forest plot showing all-cause death, MI, stroke. MI = myocardial infarction.

All-cause death outcomes, for both long-term (OR: 1.10, 95% CI: 0.95–1.24, *P* = .3) and short-term (OR: 0.95, 95% CI: 0.63–1.26, *P* = .75) follow-up, were comparable among RA patients and non-RA patients with PCI (Fig. 1).

Regarding MI, the long-term outcome was significantly higher and associated with RA patients in the PCI group (OR: 1.08, 95% CI: 1.01–1.16, *P* = .04), whereas the short-term outcome was comparable (OR: 0.91, 95% CI: 0.45–1.36, *P* = .71) (Fig. 1).

For stroke, the long-term outcome was significantly higher and associated with RA patients in the PCI group (OR: 1.09, 95% CI: 1.07–1.11, *P* < .001). The short-term outcome was comparable (OR: 0.81, 95% CI: 0.6–1.02, *P* = .07) (Fig. 1).

**Figure 2.**
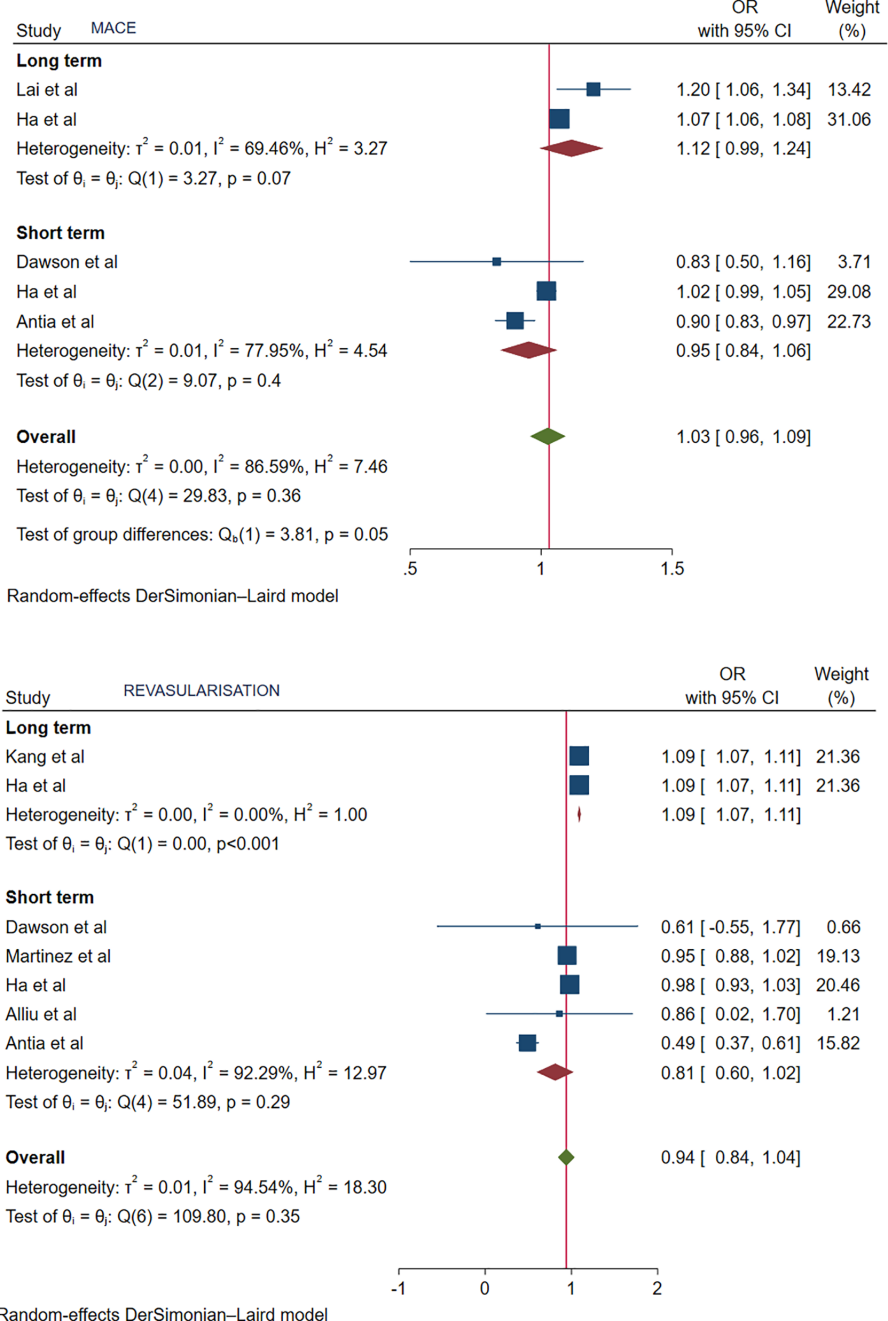
Forest plot showing MACE, and repeat revascularization. MACE = major adverse cardiovascular events.

For MACE, both long-term (OR: 1.12, 95% CI: 0.99–1.24, *P* = .07) and short-term (OR: 0.95, 95% CI: 0.84–1.06, *P* = .4) outcomes were comparable between RA and non-RA patients with PCI (Fig. 2).

For repeat revascularization, the long-term outcome was higher and significantly associated with RA patients in the PCI group (OR: 1.09, 95% CI: 1.07–1.11, *P* < .001), whereas the short-term outcome was comparable (OR: 0.81, 95% CI: 0.6–1.02, *P* = .29) (Fig. 2).

### 3.3. Publication bias and sensitivity analysis

A funnel plot using the trim and fill module showed no evidence of publication bias for each outcome in both long-term and short-term analyses (File S1, Supplemental Digital Content, https://links.lww.com/MD/P957). Sensitivity analysis did not show a significant difference, even after removing each individual study (File S2, Supplemental Digital Content, https://links.lww.com/MD/P957).

## 4. Discussion

This study aimed to investigate the short-term and long-term outcomes in RA patients following PCI by systematically reviewing the literature and conducting a meta-analysis. Our findings indicate that the risk of MACE was similar in patients with and without RA in both short-term (OR: 0.95, 95% CI: 0.84–1.06, *P* = .4) and long-term (OR: 1.12, 95% CI: 0.99–1.24, *P* = .07) follow-ups. Although not statistically significant in this study, the trend may suggest a potential increase in the long-term risk of MACE in RA patients, which is consistent with prior studies.^[17,22,26]^

In terms of individual outcomes, the risk of MI was not significantly different in patients with and without RA in the short term (OR: 0.91, 95% CI: 0.45–1.36, *P* = .71). However, the long-term outcomes demonstrated significantly higher odds of MI in RA patients (OR: 1.08, 95% CI: 1.01–1.16, *P* = .04). Similarly, patients with RA had a significantly increased long-term risk of stroke (OR: 1.09, 95% CI: 1.07–1.11, *P* < .001), with no significant difference in short-term risk (OR: 0.81, 95% CI: 0.6–1.02, *P* = .07). Repeat revascularization short-term outcomes were comparable (OR: 0.81, 95% CI: 0.6–1.02, *P* = .29), whereas RA patients had significantly higher odds of repeat revascularization in the long term (OR: 1.09, 95% CI: 1.07–1.11, *P* < .001). All-cause death outcomes, both short-term (OR: 0.95, 95% CI: 0.63–1.26, *P* = .75) and long-term (OR: 1.10, 95% CI: 0.95–1.24, *P* = .3), were comparable between RA and non-RA patients.

It is noteworthy that the rates of all-cause death and short-term cardiovascular events were not significantly different between patients with or without RA. This suggests that despite the increased long-term risk of specific adverse outcomes, the short-term prognosis remains comparable. Furthermore, the rates of all-cause death and MACE were not found to be significantly different between patients with or without RA, implying that the overall outcomes are still comparable despite the increased risk of certain cardiovascular events in RA patients.

Long-term inflammation related to RA may explain the significantly increased risk of MI and stroke in the long term, which is not observed in the short term. Chronic systemic inflammation in RA plays a vital role in the progression of atherosclerosis. Elevated levels of pro-inflammatory cytokines, such as tumour necrosis factor-alpha (TNF-alpha), interleukin-1 (IL-1), and interleukin-6 (IL-6), contribute to endothelial dysfunction by reducing the bioavailability of nitric oxide, increasing oxidative stress, and impairing vasodilation. These changes facilitate plaque development and subsequent instability by promoting white blood cell adhesion, foam cell formation, and smooth muscle proliferation. Systemically, these inflammatory mediators also contribute to dyslipidemia and insulin resistance, further compounding the inherently increased cardiovascular risk.^[2,6]^

Interestingly, RA has also been associated with impairment in systolic and diastolic cardiac function. These subclinical changes predispose patients with RA to heart failure with preserved or reduced ejection fraction, thus indirectly contributing to the increased predisposition of patients with RA to ischemic events in the long term.^[2,6]^

The inherent increased risk of cardiovascular events in patients with RA led to The European League Against Rheumatism (EULAR) task force recommending cardiovascular risk assessments in RA patients every 2 to 3 years or more frequently with changes in disease activity and cardiovascular risk.^[27]^ This recommendation was modified to once every 5 years after the task force reconvened in 2015.^[28]^ It has also been recommended that cardiovascular risk scores be multiplied by 1.5 in patients with RA meeting at least 2 of 3 criteria (disease duration of more than 10 years, presence of rheumatoid factor or anti-cyclic citrullinated peptide (anti-CCP) positivity, and presence of certain extra-articular manifestations).^[27]^ Studies also document a decreased risk of cardiovascular events in general with the use of Disease-Modifying Antirheumatic Drugs (DMARDs),^[29]^ especially methotrexate and biologics (TNF-alpha inhibitors), and an increased risk with prolonged corticosteroid use.^[30]^ The inherent increased risk of cardiovascular events in patients with RA led to The European League against Rheumatism guidelines (EULAR) task force recommending cardiovascular risk assessments in RA patients every 2 to 3 years or more frequently with change in disease activity and cardiovascular risk,^[27]^ which was modified to once every 5 years after the task force reconvened in 2015.^[28]^ It has also been recommended that cardiovascular risk scores be multiplied by 1.5 in patients with RA meeting at least 2 of 3 criteria (disease duration of more than 10 years, rheumatoid factor or anti-CCP positivity and presence of certain extra-articular manifestations).^[27]^ Studies also document decreased risk of cardiovascular events in general with use of DMARDs, especially methotrexate^[29]^ and biologics (TNF-alpha inhibitors), and increased risk with prolonged corticosteroid use.^[30]^

The strong association between RA and increased cardiovascular risk is likely implicated in the findings of our study. Management by a multi-disciplinary team of rheumatologists, cardiologists, and primary care providers may therefore significantly improve outcomes and help counteract the increase in long-term risk post-PCI. The findings of our study emphasize the importance of long-term follow-up, regular monitoring, and patient education to help identify and treat early signs of MI and stroke effectively.

This study highlights the necessity of collaborative efforts and dedicated risk stratification strategies that account for activities of daily living, medication, and vascularization status to stratify RA patients undergoing PCI based on the risk of adverse long-term cardiovascular outcomes. Based on their risk profile, personalized management protocols and regular follow-ups can be recommended. Moreover, secondary prevention initiatives can also be planned to reduce the instance of repeat revascularization through early diagnosis, lifestyle modifications, and managing comorbidities.

## 5. Limitation

The main limitation of this study was the heterogeneity among the included studies. Previously conducted studies often lacked significant contributions to long-term data, which could influence the results. Additionally, some areas of the studies presented data irrelevant to our present scope. This heterogeneity directly influences the weight of the different studies in the meta-analysis.

## 6. Conclusion

PCI in RA patients provides short-term benefits comparable to non-RA patients, but it presents significantly higher long-term risks of repeat revascularization, MI, and stroke events. Thus, comprehensive cardiovascular risk management, personalized care plans, and collaborative efforts are necessary to improve long-term outcomes in this vulnerable patient population.

## Author contributions

**Conceptualization:** Sulochana Khadka.

**Data curation:** Sulochana Khadka, Bibek Timilsina, Akshata Ammembal, Faisal Chowdhury, Karun Suwal, Bibek Shrestha.

**Formal analysis:** Bishow Nath Adhikari, Bimarsh Acharya.

**Investigation:** Rabin Baniya, Sushil Gyawali, Sonali Kumari Shah, Rosna Thapa.

**Writing – original draft:** Sulochana Khadka, Bibek Timilsina, Akshata Ammembal, Faisal Chowdhury, Karun Suwal, Bibek Shrestha.

**Writing – review & editing:** Faisal Chowdhury, Bishow Nath Adhikari, Bimarsh Acharya, Rabin Baniya, Sushil Gyawali, Sonali Kumari Shah, Rosna Thapa.

## Supplementary Material


