# Supplementary material for: Short-term and long-term outcomes in rheumatoid arthritis patients following percutaneous coronary intervention: A systematic review and meta-analysis
Source: Medicine (Baltimore). 2025 Sep 12;104(37):e44458. doi: 10.1097/MD.0000000000044458 (PMC12440515; doi:10.1097/MD.0000000000044458)
Supplement: Supplementary file 1 [file medi-104-e44458-s001.pdf]

Supplementary file 1: Funnel Plots evaluating model biases

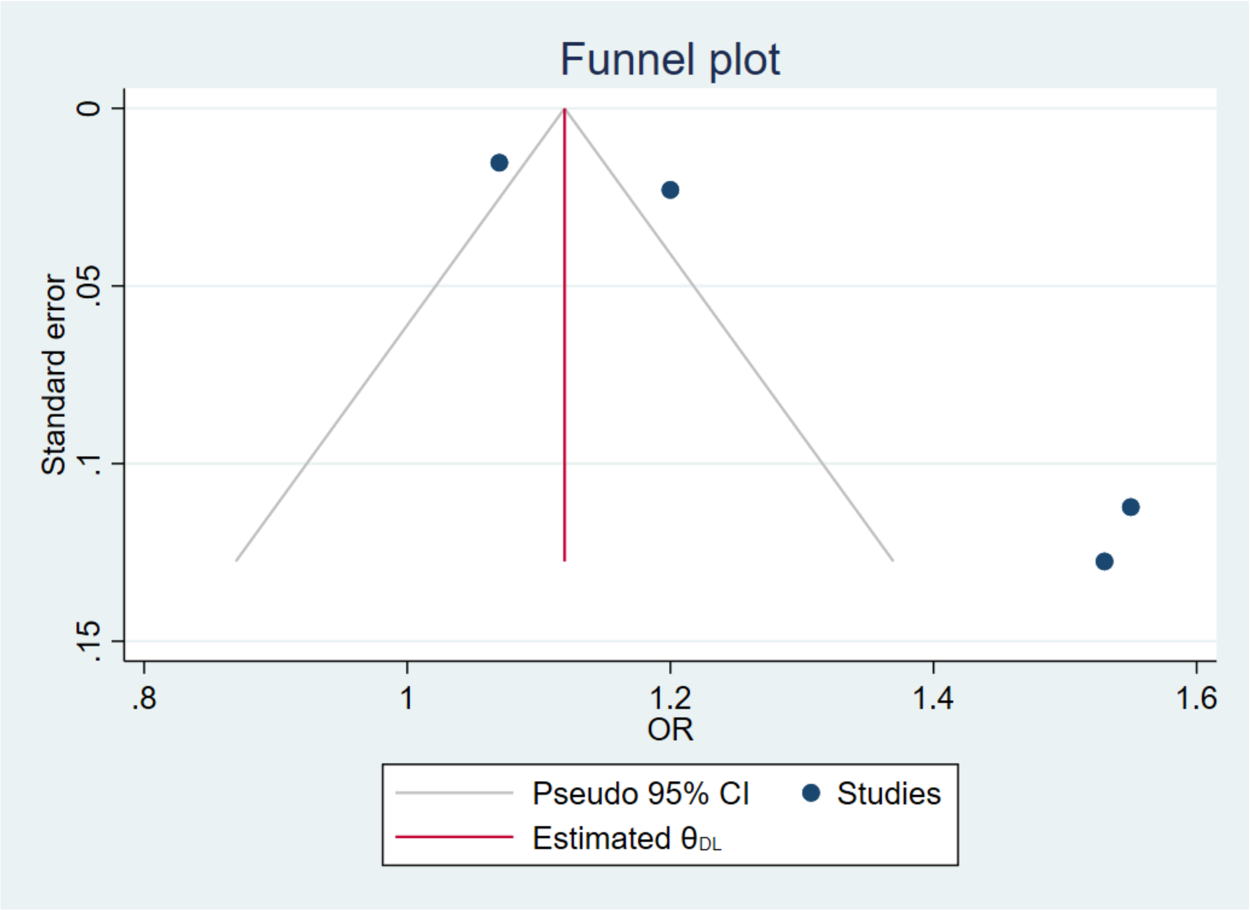

Figure: Funnel plot for the meta-analysis of four studies on long-term all-cause mortality

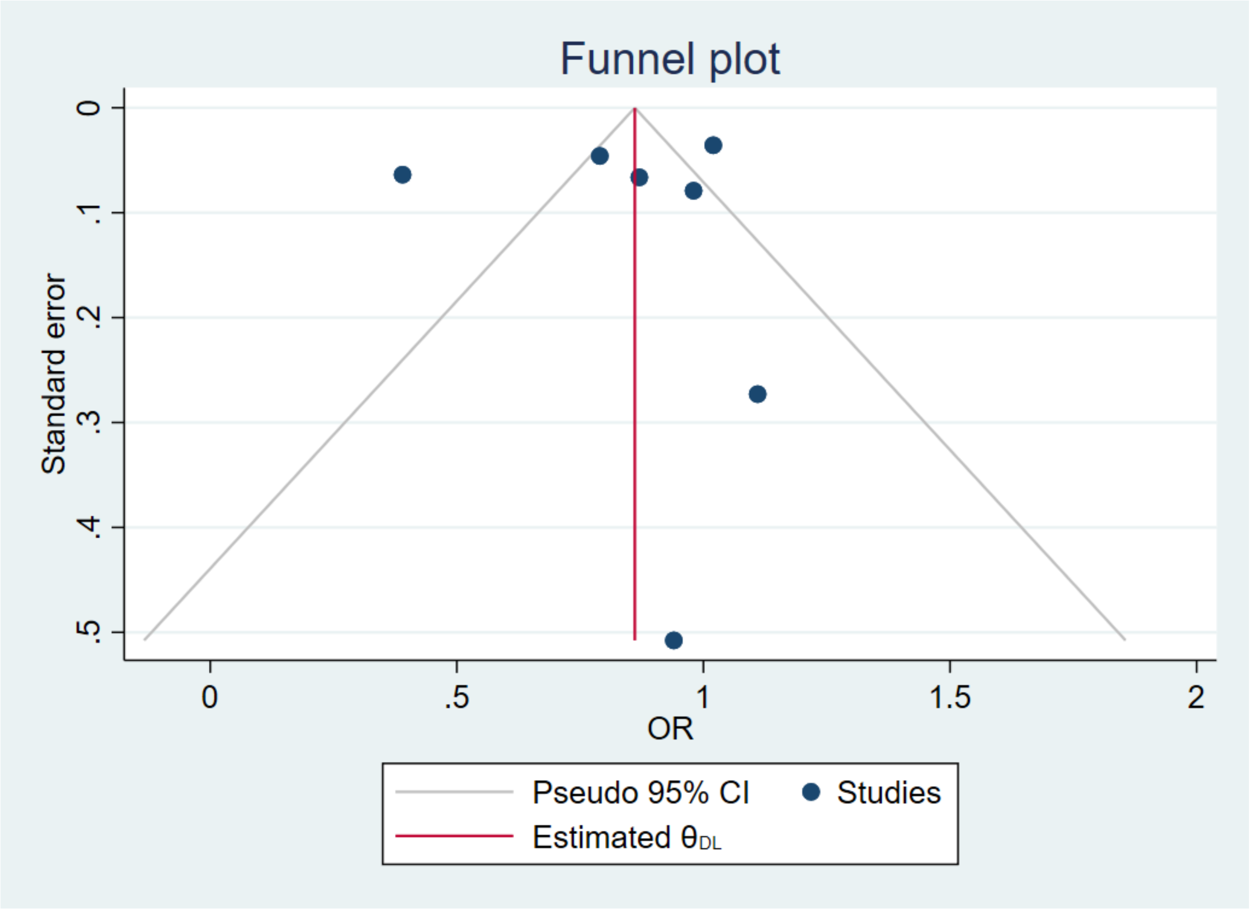

Figure: Funnel plot for the meta-analysis of seven studies on short-term all-cause mortality

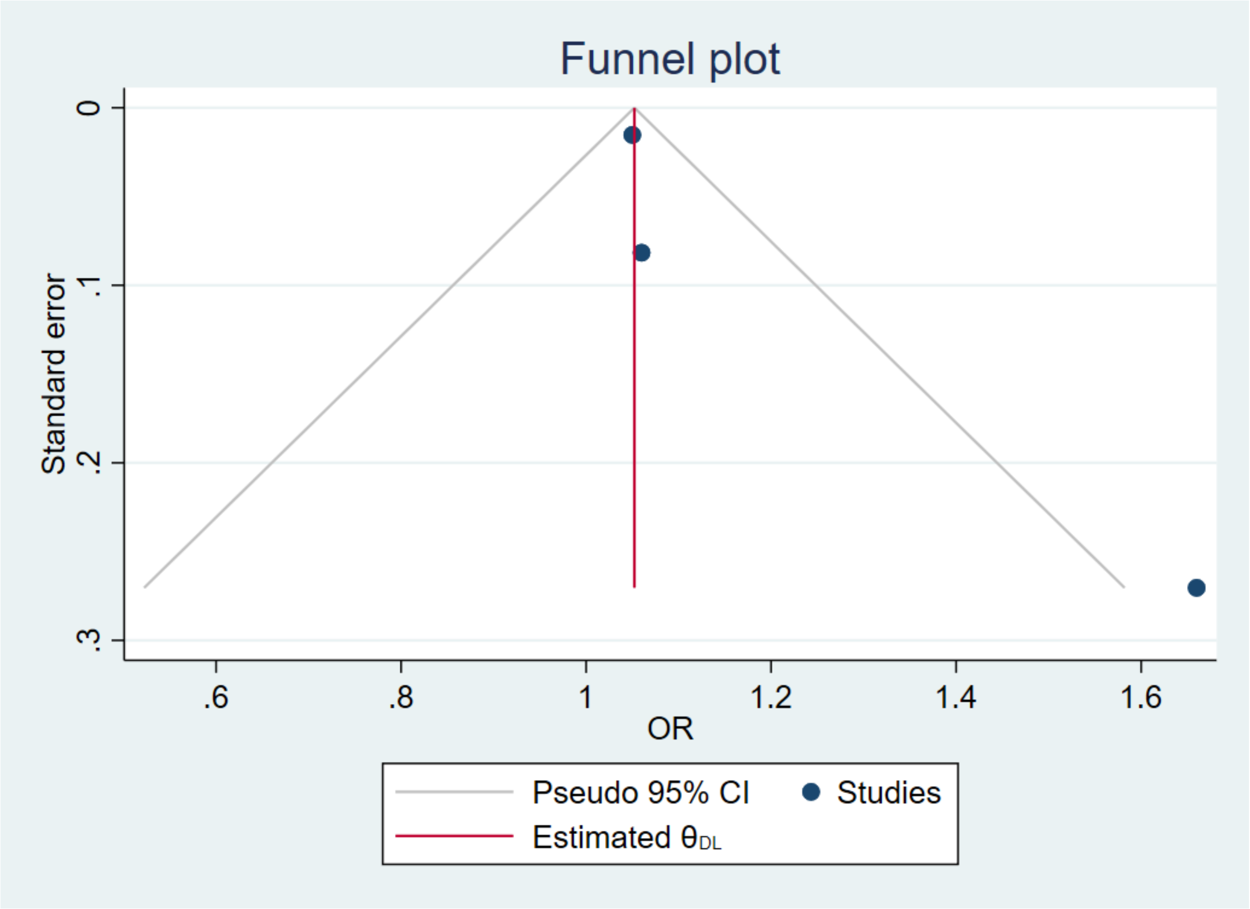

Figure : Funnel plot for the meta-analysis of three studies on long-term revascularization

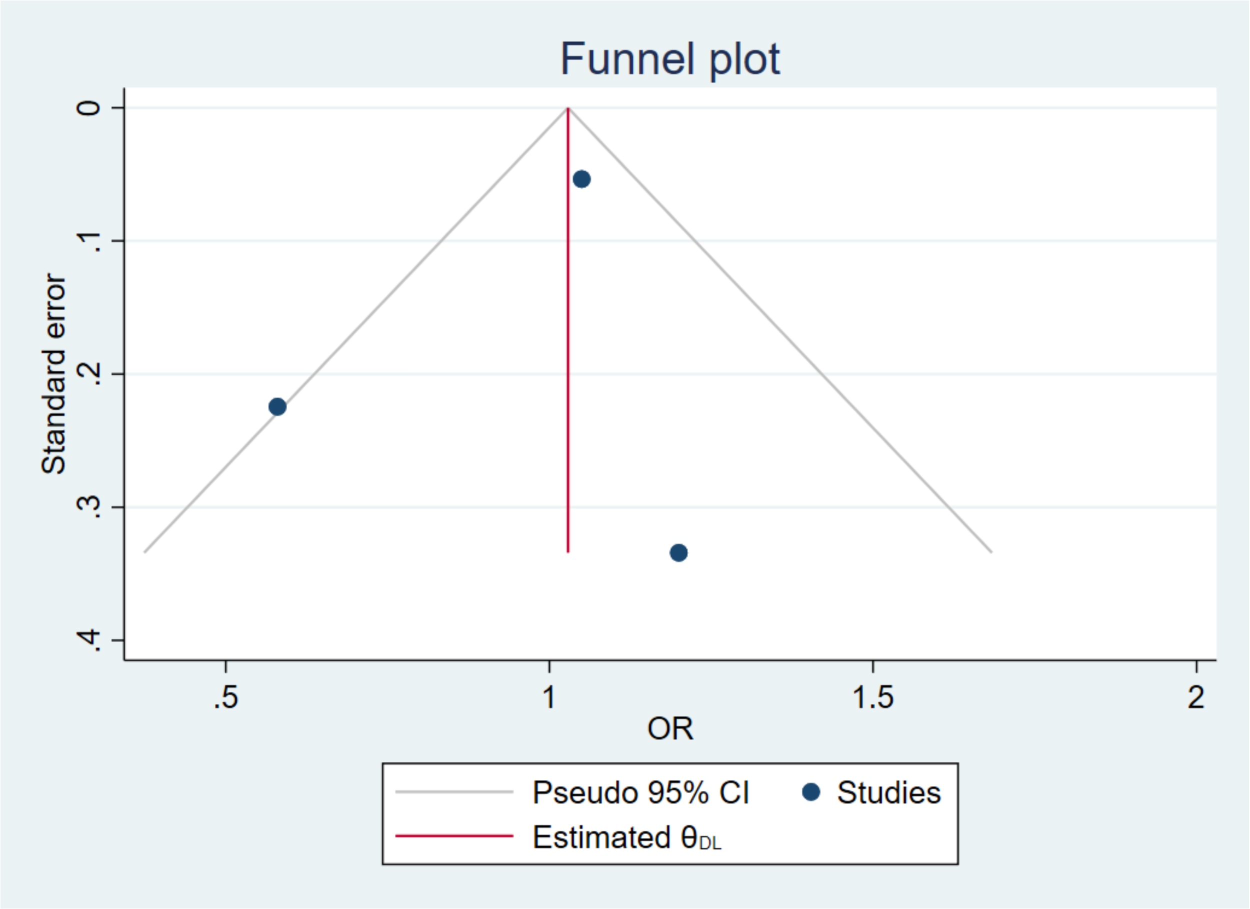

Figure: Funnel plot for the meta-analysis of three studies on short-term revascularization

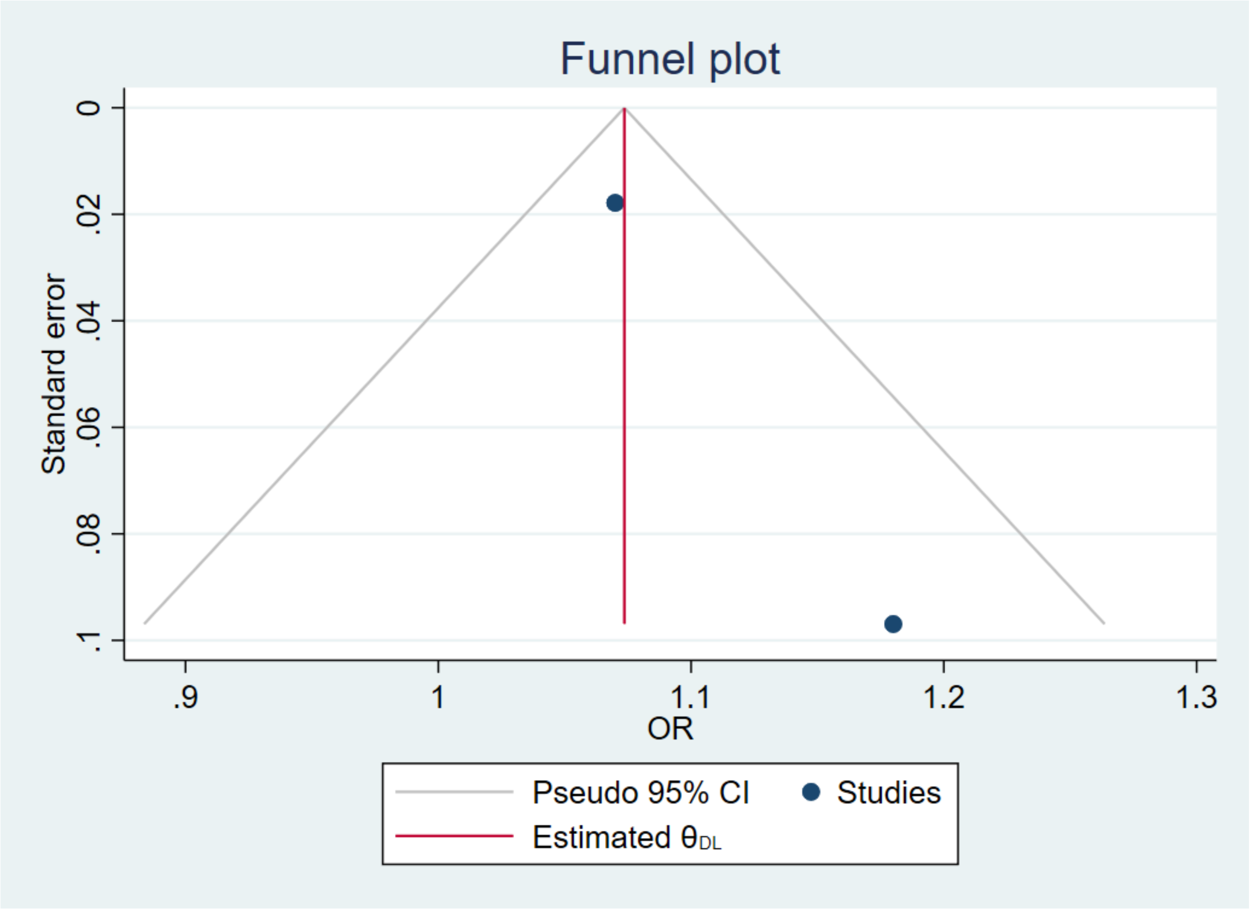

Figure: Funnel plot for the meta-analysis of two studies on long-term MI

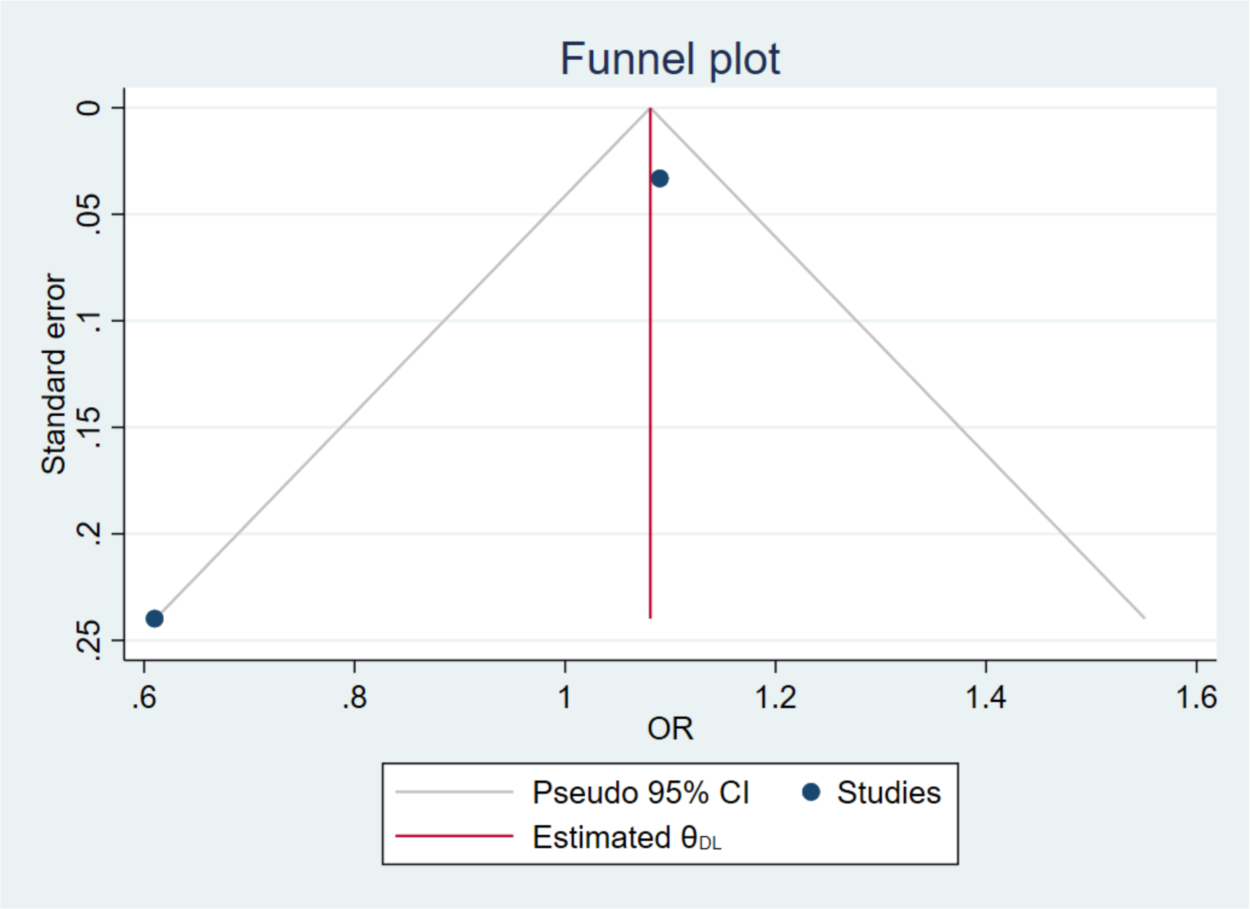

Figure: Funnel plot for the meta-analysis of two studies on short-term MI

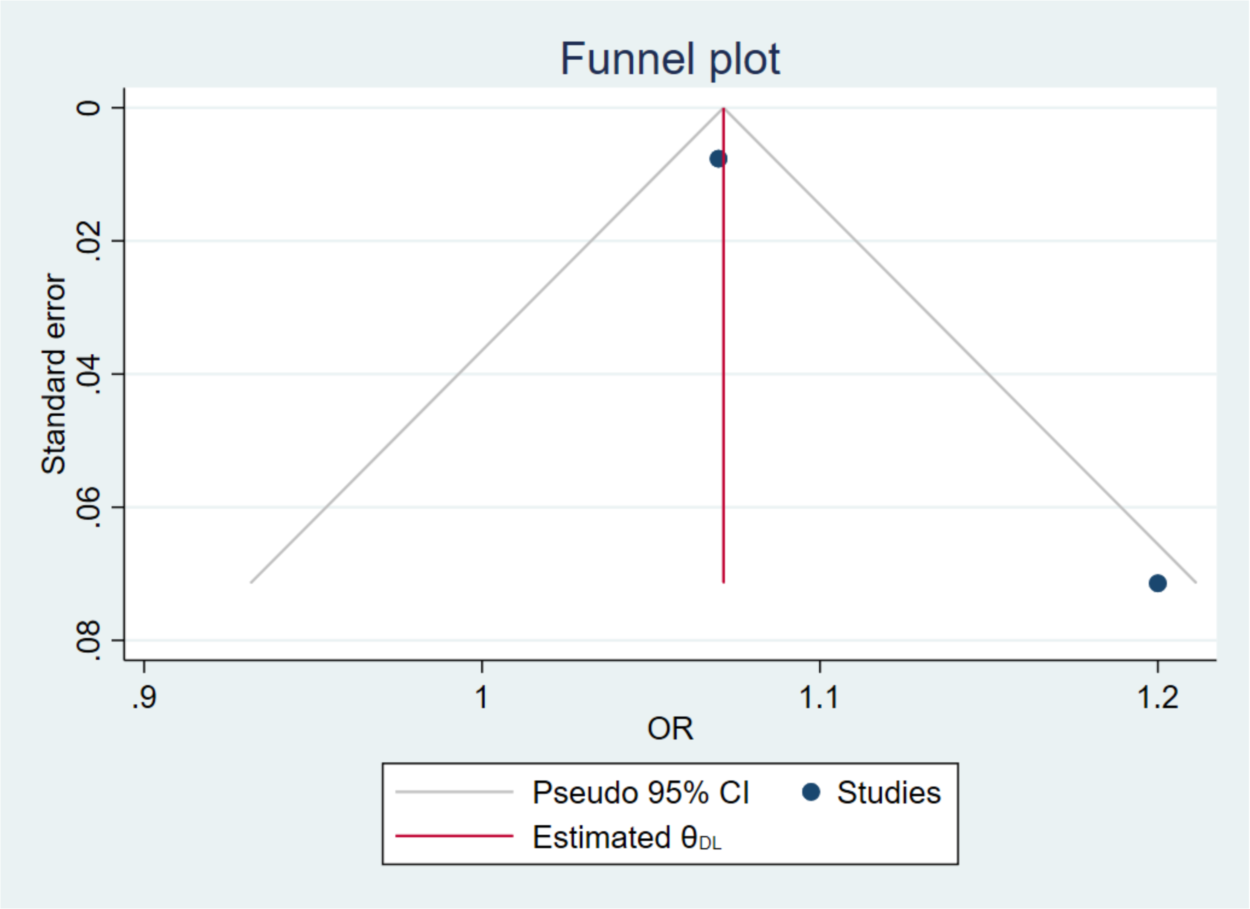

Figure: Funnel plot for the meta-analysis of two studies on long-term MACE

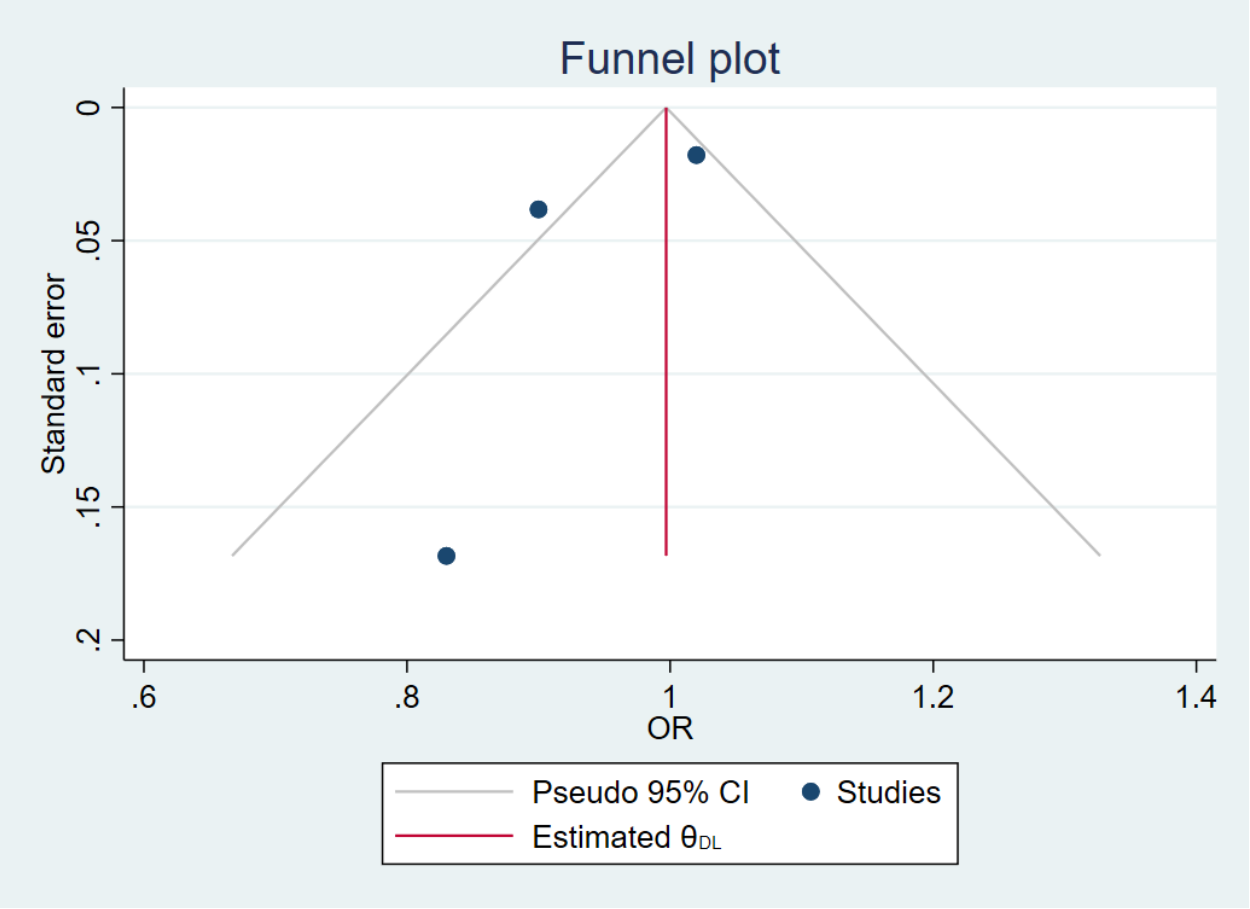

Figure: Funnel plot for the meta-analysis of three studies on short-term MACE

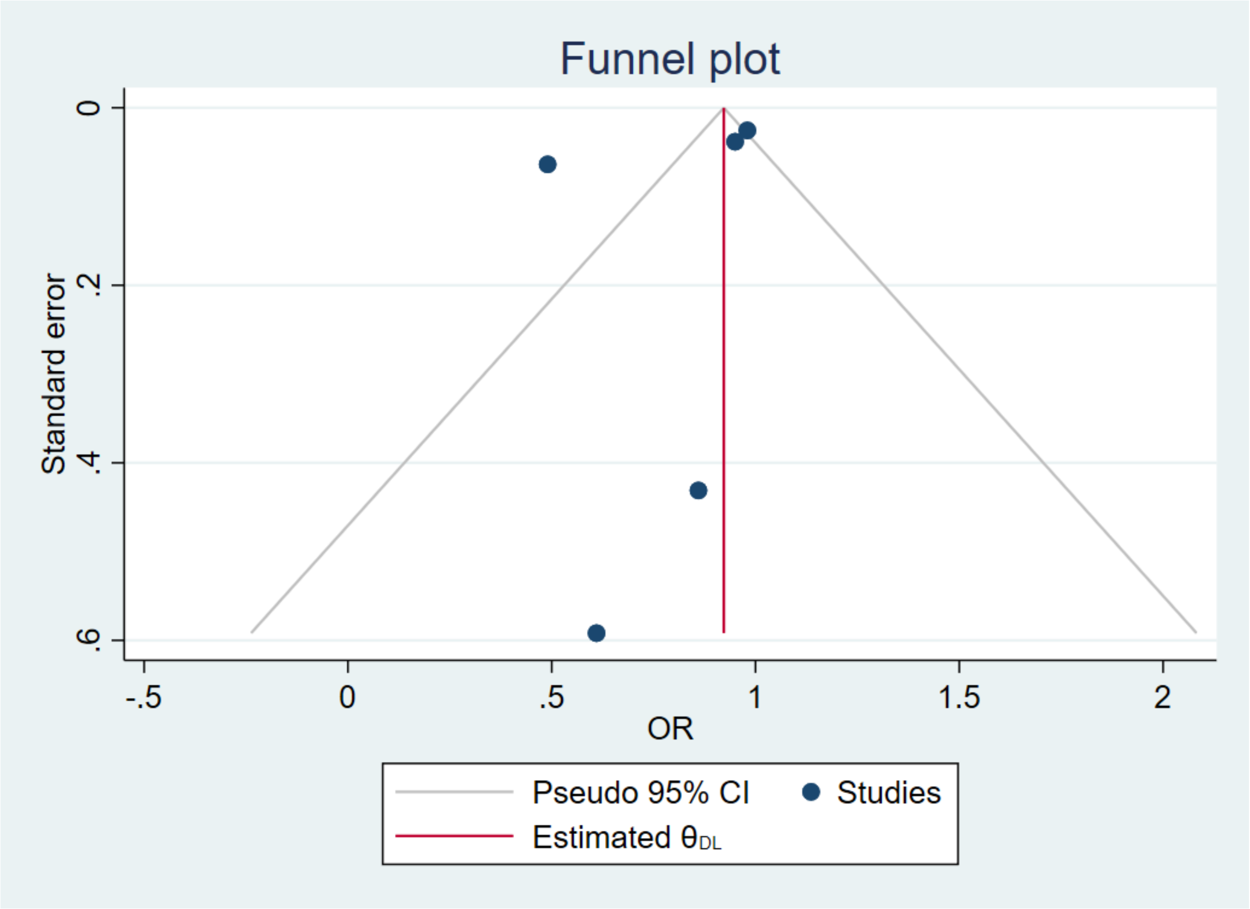

Figure: Funnel plot for the meta-analysis of five studies on long-term stroke

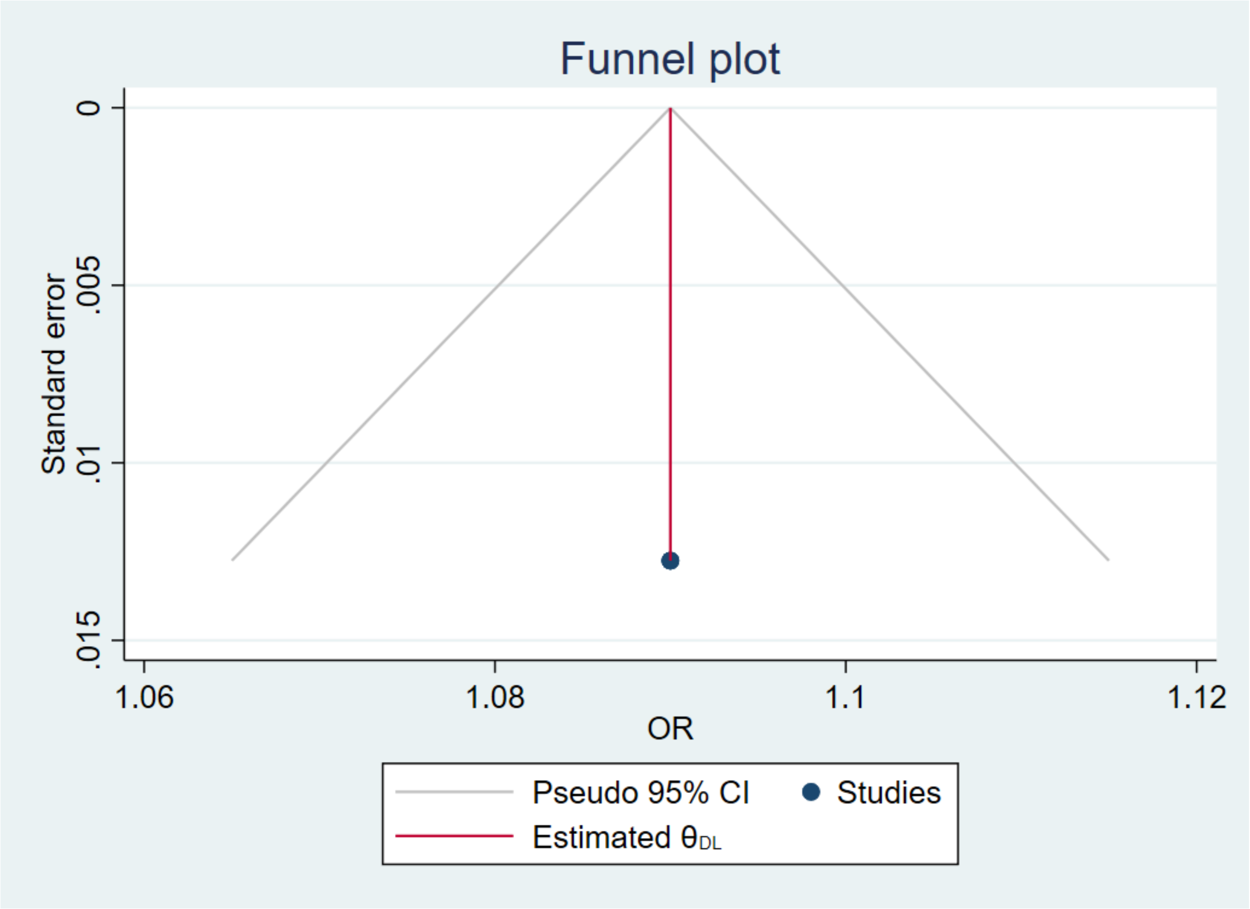

Figure: Funnel plot for the meta-analysis of one on short-term stroke

Supplementary file 2: Leave-one-out sensitivity analysis for long- and short-term mortality

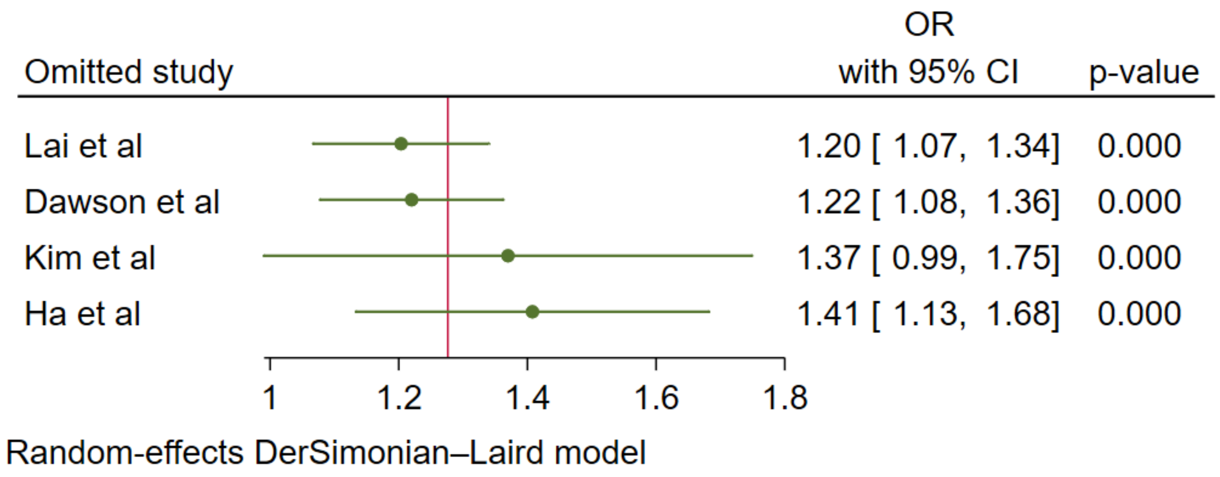

Figure : Leave-one-out sensitivity analysis for long term follow-up

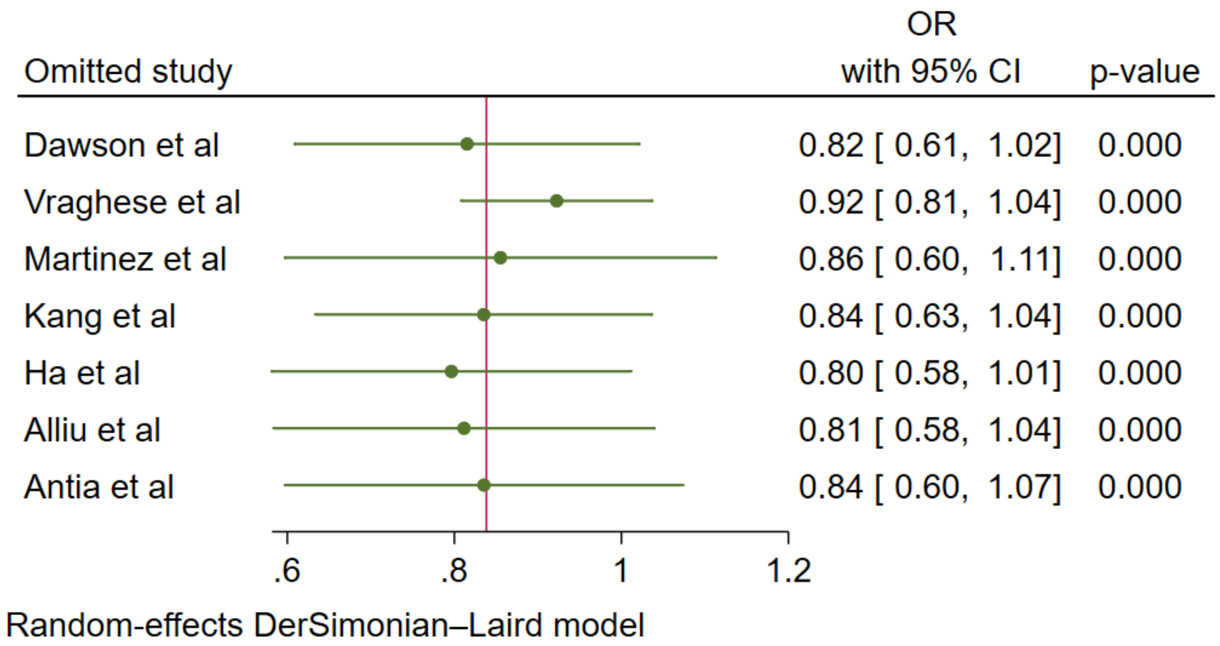

Figure : Leave-one-out sensitivity analysis for short term follow-up

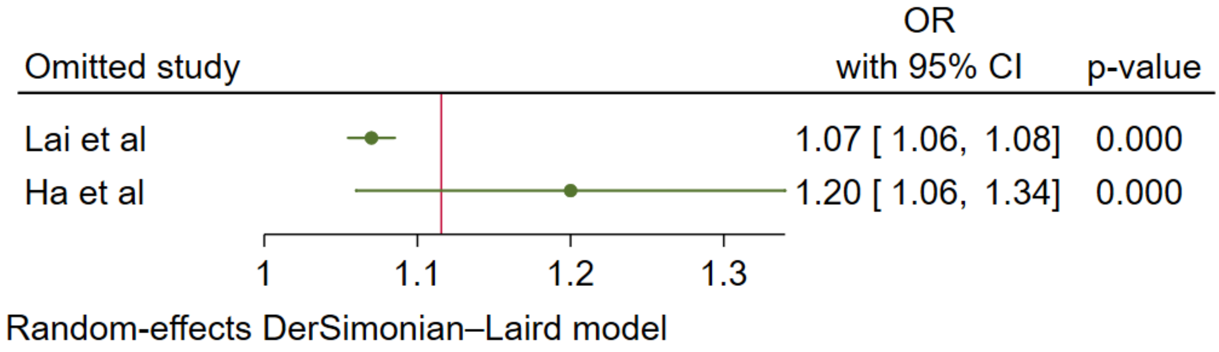

Figure : Leave-one-out sensitivity analysis for long term MACE

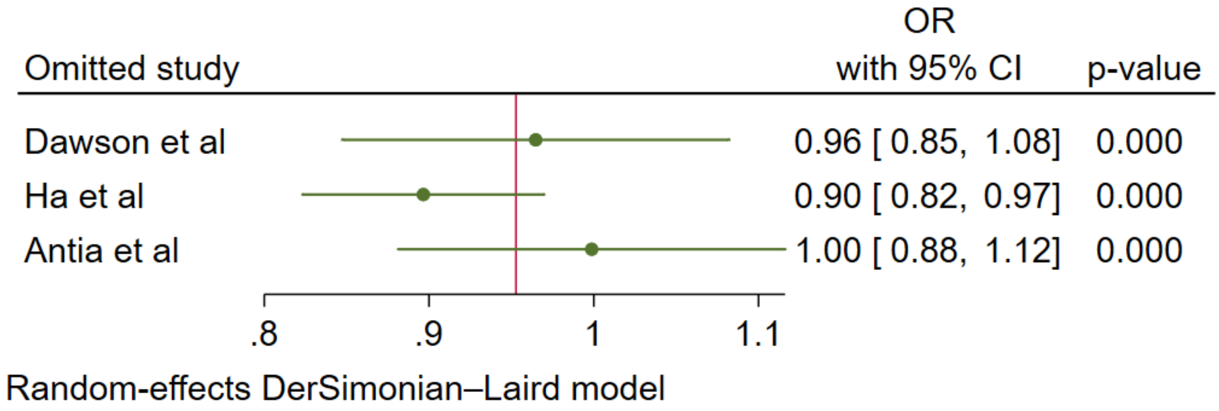

Figure : Leave-one-out sensitivity analysis for short term MACE

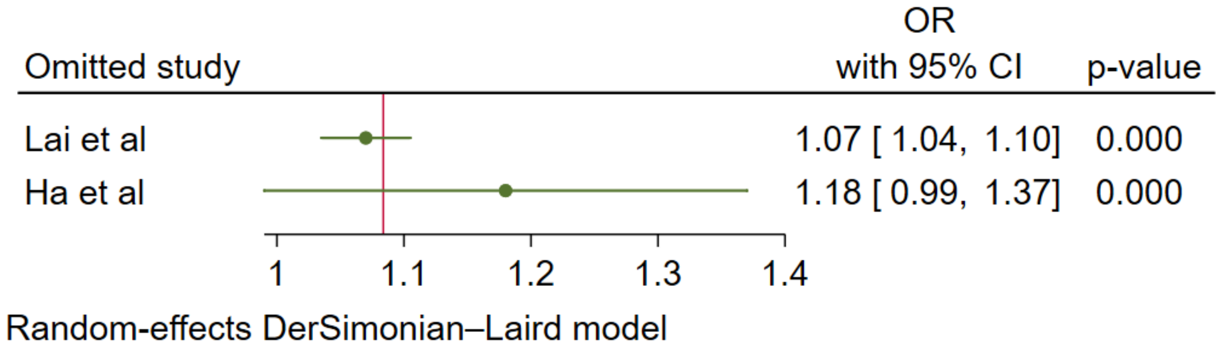

Figure : Leave-one-out sensitivity analysis for long term MI

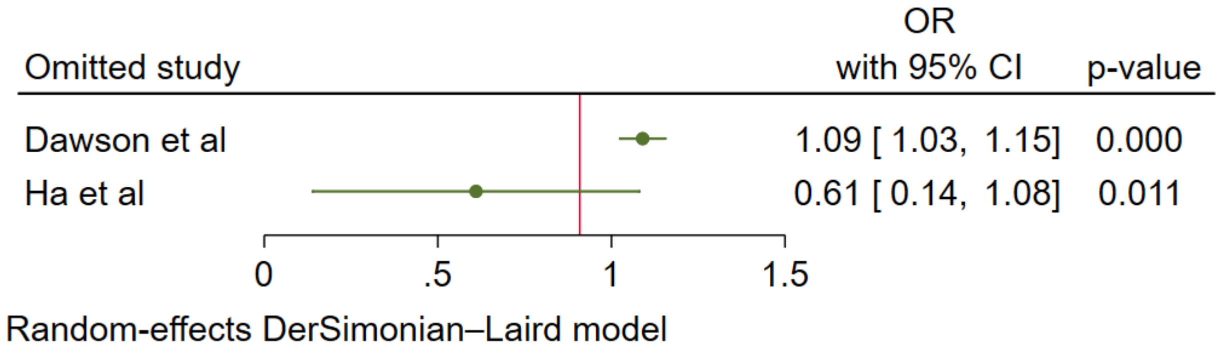

Figure : Leave-one-out sensitivity analysis for short term MI

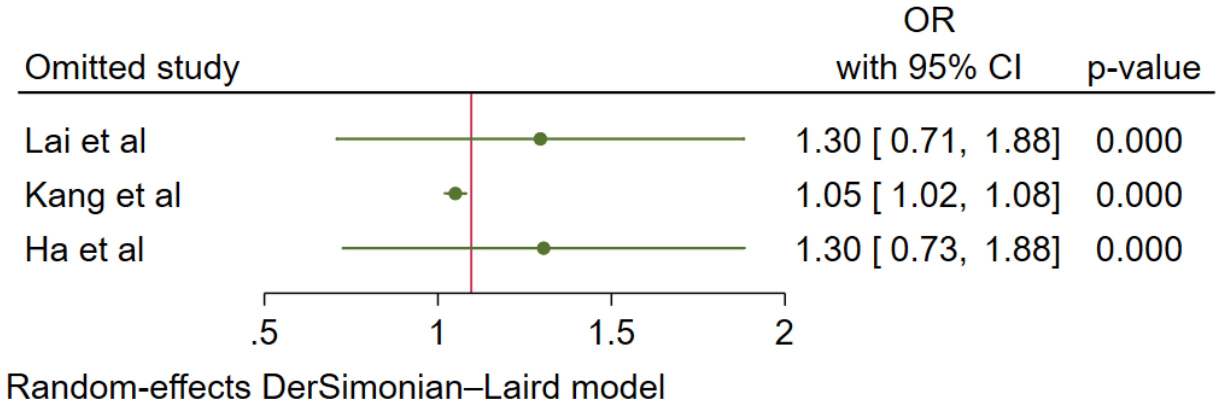

Figure : Leave-one-out sensitivity analysis for long term revascularization

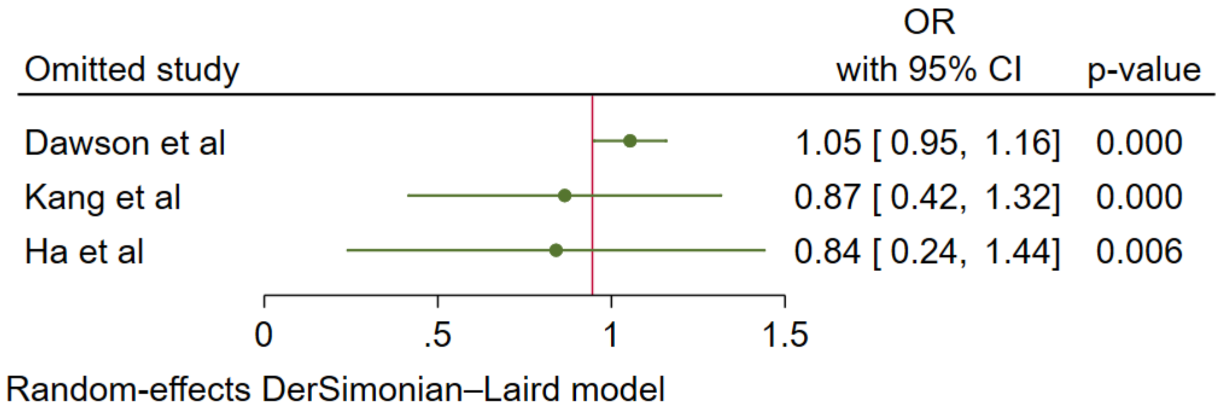

Figure : Leave-one-out sensitivity analysis for short term revascularization

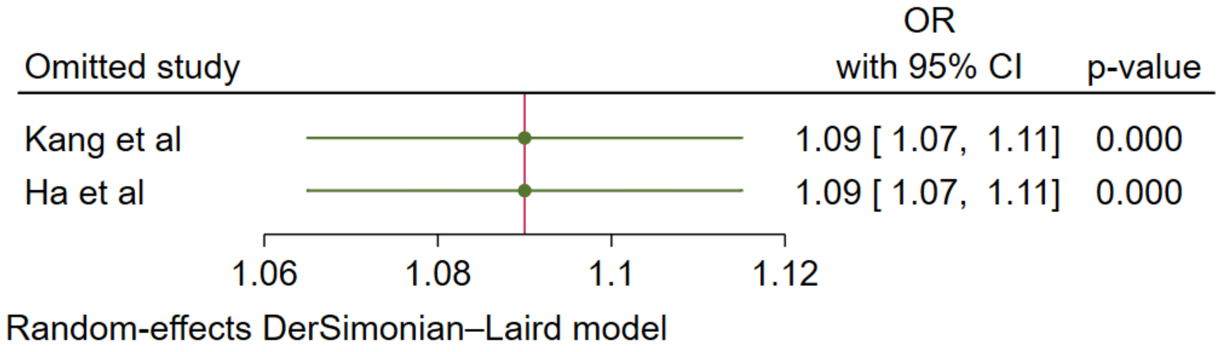

Figure : Leave-one-out sensitivity analysis for long term stroke

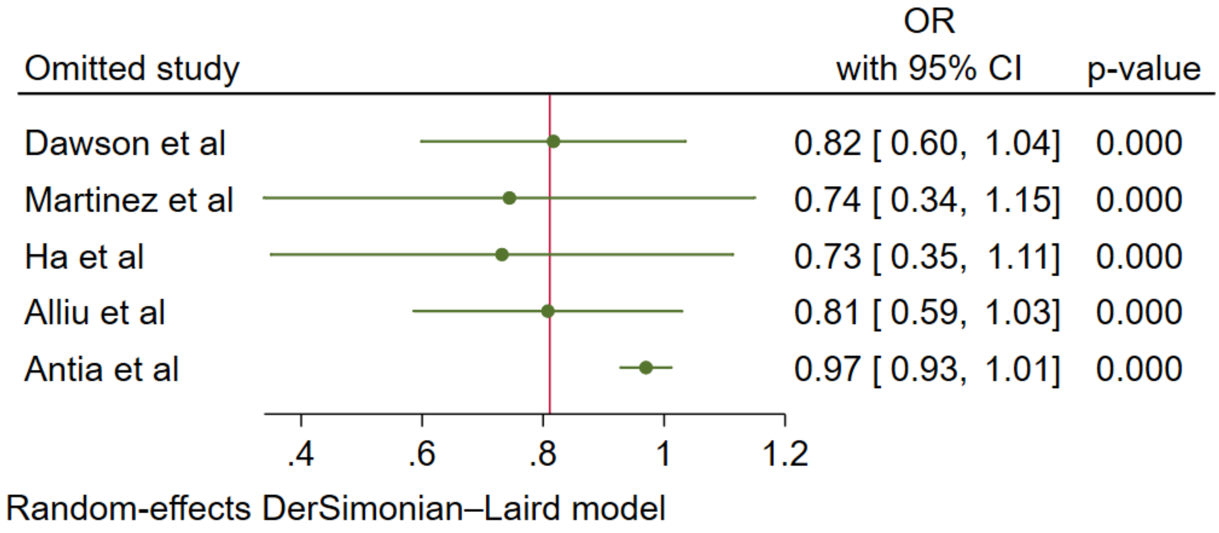

Figure : Leave-one-out sensitivity analysis for short term stroke

Supplemental File 3: Search Strategy for Systematic Review

((("Arthritis, Rheumatoid"[Mesh]) OR (Rheumatoid Arthritis[tiab]) OR (RA[tiab]) OR (Inflammatory Arthritis[tiab] AND (Rheumatoid[tiab] OR Seropositive[tiab] OR Seronegative[tiab])))

AND

(("Percutaneous Coronary Intervention"[Mesh]) OR ("Angioplasty, Transluminal, Percutaneous Coronary"[Mesh]) OR ("Stents"[Mesh]) OR ("Drug-Eluting Stents"[Mesh]) OR ("Coronary Artery Bypass"[Mesh]) OR ("Myocardial Revascularization"[Mesh]) OR ("Myocardial Ischemia"[Mesh]) OR ("Cardiomyopathies"[Mesh]) OR ("Ventricular Dysfunction, Left"[Mesh]) OR ("Heart Failure, Systolic"[Mesh]) OR (Percutaneous Coronary Intervention*[tiab]) OR (PCI[tiab]) OR (Coronary Angioplasty[tiab]) OR (Coronary Stent*[tiab]) OR (Coronary Artery Bypass Graft*[tiab]) OR (CABG[tiab]) OR (Myocardial[tiab] AND Revascularization[tiab]) OR (Coronary Revascularization[tiab]) OR (Revascularization[tiab] AND (Coronary[tiab] OR Cardiac[tiab] OR Myocardial[tiab])) OR (Ischemic Cardiomyopathy[tiab] OR Ischaemic Cardiomyopathy[tiab]) OR (Coronary Intervention*[tiab]) OR (Left Ventricular Systolic Dysfunction[tiab]) OR (LVSD[tiab]) OR (Ejection Fraction[tiab] AND (Low[tiab] OR Reduced[tiab] OR Impaired[tiab] OR Dysfunction[tiab]))))
