# Supplementary material for: Short-term and long-term outcomes in rheumatoid arthritis patients following percutaneous coronary intervention: A systematic review and meta-analysis
Source: Medicine (Baltimore). 2025 Sep 12;104(37):e44458. doi: 10.1097/MD.0000000000044458 (PMC12440515; doi:10.1097/MD.0000000000044458)
Supplement: Supplementary file 2 [file medi-104-e44458-s002.docx]

Supplementary Table 1: Risk of Bias of the included study

| Study | Study Design | Country | NOS Selection | NOS Comparability | NOS Outcome | Total NOS |
| --- | --- | --- | --- | --- | --- | --- |
| Lai et al. (2015) | Retrospective Cohort Study | Taiwan | 3 | 2 | 2 | 7 |
| Dawson et al. (2020) | Cohort Study | Australia | 3 | 2 | 2 | 7 |
| Varghese et al. (2009) | Cross-Sectional Study | United States of America | 3 | 2 | 2 | 7 |
| Kim et al. (2023) | Retrospective Cohort Study | South Korea | 3 | 2 | 3 | 8 |
| Martinez et al. (2020) | Retrospective Cohort Study | United States of America | 3 | 2 | 2 | 7 |
| Kang et al. (2012) | Retrospective Cohort Study | Taiwan | 3 | 2 | 2 | 7 |
| Ha et al. (2023) | Retrospective Cohort Study | South Korea | 3 | 2 | 2 | 7 |
| Samson Alliu et al. (2019) | Retrospective Cohort Study | United States of America | 3 | 2 | 2 | 7 |
| Antia et al. (2024) | Retrospective Cohort Study | United States of America | 3 | 2 | 2 | 7 |
